# Amyloidosis and risk of psychiatric disorders-insights from a real-world nationwide registry^☆^

**DOI:** 10.1016/j.clinsp.2026.101208

**Published:** 2026-10-16

**Authors:** Wei-Syun Hu, Po-Hsiang Liao, Cheng-Li Lin, Chih-Yang Huang

**Affiliations:** a School of Medicine, College of Medicine, China Medical University, Taichung, Taiwan; b Division of Cardiovascular Medicine, Department of Medicine, China Medical University Hospital, Taichung, Taiwan; c Center for Drug Research and Development, Chang Gung University of Science and Technology, Taoyuan, Taiwan; d Management Office for Health Data, China Medical University Hospital, Taichung, Taiwan; e Department of Precision Chinese Herbal Medicine and Molecular Medicine Research, E-Da Cancer Hospital, I-Shou University, Kaohsiung, Taiwan; f Department of Precision Chinese Herbal Medicine and Molecular Medicine Research, E-Da Hospital, I-Shou University, Kaohsiung, Taiwan; g School of Chinese Medicine for Post Baccalaureate, I-Shou University, Kaohsiung, Taiwan; h Department of Medical Research, China Medical University Hospital, China Medical University, Taichung, Taiwan; i Superintendent's Office, Hualien Tzu Chi Hospital, Buddhist Tzu Chi Medical Foundation, Hualien, Taiwan; j Office of Research & Development, Asia University, Taichung, Taiwan; k Department of Nutritional Science, Fu Jen Catholic University, New Taipei, Taiwan

**Keywords:** Amyloidosis, Psychiatric disorder, Risk

## Abstract

**Objective:**

This is a National Health Insurance (NHI) database research investigating the association between amyloidosis and psychiatric disorders risk.

**Methods:**

The amyloidosis cohort was identified from insured individuals with amyloidosis, who were newly diagnosed from 2009 to 2019. Both cohorts were followed up and the subsequent psychiatric disorder was the major outcome event for the research. The multivariable model were used to examine the effect of amyloidosis and risk factor on the risk of developing psychiatric disorder.

**Results:**

The psychiatric disorder incidence was higher in the amyloidosis than in the comparison cohort (11.3 vs. 10.3 per 1000 person-years, crude HR = 1.10, 95% CI 1.02–1.19), with adjusted HR (aHR) of 1.26 (95% CI 1.16–1.36). After controlling for the confounders, amyloidosis cohort had a 1.38- fold higher risk of senile and pre-senile organic psychotic conditions (95% CI 1.22‒1.57), 1.26-fold higher risk of bipolar affective disorder (95% CI 1.10‒1.45), and 1.16-fold higher risk of other organic psychotic conditions than comparison cohort (95% CI 1.01-1.32).

**Conclusions:**

The authors found that compared to a matched amyloidosis naïve cohort, psychiatric disorders risk was significantly higher in patients with amyloidosis. Among different psychiatric disorders, risks of senile and pre-senile organic psychotic conditions, bipolar affective disorder and other organic psychotic conditions were higher among people with amyloidosis.

## Highlights

• This article analyzed amyloidosis population and showed significant worse psychiatric outcome.
• The sample size of each group is big enough comparing to prior studies.
• Among different psychiatric disorders, several conditions were higher among amyloidosis.

## Introduction

Amyloidosis is an extremely rare extracellular protein deposit disorder involving multiple systems leading to cellular, tissue and organ destruction.^1^^,^^2^ Amyloidosis has a huge spectrum of disease severity and clinical outcome is quite different according to cause, severity, underlying mechanisms, or organs involvement.^1^, ^2^, ^3^, ^4^ A delayed diagnosis of amyloidosis is often encountered since clinical presentations are usually vague and atypical unless highly suspicion. Once a diagnosis has been made, target medications and chemotherapy or even stem cell-based therapy are possible solutions. The impact of psychiatric disorders on amyloidosis has been studied previously;^5^^,^^6^ the knowledge gaps are going to be filled by this study since we are investigating this issue with different approach in a real-world setting. In the current investigation, we would provide an observation assessing data from the Taiwan National Health Insurance Research Database (NHIRD) in a retrospective cohort study implementing matching analysis to strengthen the validity and robustness of the results, aiming to explore whether people with amyloidosis tend to have psychiatric disorders more frequently and explored the different at-risk subgroups.

## Methods

### Data source

The analyzed data in this study were retrieved from Taiwan NHIRD from January 2009 through December 2020. Since 1995, the National Health Insurance (NHI) program has been implemented, with more than 99% of the Taiwan population enrolled.^7^^,^^8^ The NHIRD includes registration and claims files from all hospitals and clinics. The identification information was encrypted due to the privacy issue. The disease code in NHIRD follows the International Classification of Diseases, Ninth & Tenth Revision, Clinical Modification (ICD-9-CM & ICD-10-CM). This study was approved by the Institutional Review Board of China Medical University Hospital Research Ethics Committee (CMUH111-REC2-109(CR-1)).

### Study population

The amyloidosis cohort was identified from insured individuals with amyloidosis (ICD-9 code 277.3; ICD-10 code E85), who were newly diagnosed from 2009 to 2019. The date of amyloidosis diagnosis was designated as the index date. For each identified amyloidosis patient, one comparison were randomly selected from individuals without amyloidosis, matched by sex, age, and baseline year using propensity score. Patients younger than 20-years, those having a history of psychiatric disorder (ICD-9: 290-298; ICD 10: F01-F06, F09-F20, F22-F25, F28-F34, F53), and those with incomplete age or sex information were excluded.

### Main outcome and confounders

Both cohorts were followed up and the subsequent psychiatric disorder was the major outcome event for the research. Person-years in the follow-up period were counted for each individual until psychiatric disorder development, withdrawal from the insurance or the end of 2020. In addition to the demographic factors, the comorbidities that may be associated with psychiatric disorder were identified. These include sex, age, and comorbidities with hypertension, diabetes mellitus, hyperlipidaemia, atrial fibrillation (AF), coronary artery disease (CAD), sleep disorders, anxiety, stroke, and chronic obstructive pulmonary disease (COPD).

### Statistical analysis

We compared baseline characteristics between the amyloidosis and comparison cohorts using the Chi-Square test to examine category variables and the Student's *t*-test for continuous variables. We estimated the follow-up person-years to measure the incidence rate of psychiatric disorder, and subsequently calculated the amyloidosis to comparison cohort relative Hazard Ratios (HRs) and the 95% Confidence Interval (CI) using univariable and multivariable Cox proportional hazards regression models. The multivariable model were used to examine the effect of amyloidosis and risk factors on the risk of developing psychiatric disorder. The SAS® 9.4 software (Statistical Analysis System (SAS) Institute Inc. (SAS Institute), Cary, NC, USA) was used for data analysis. The comparisons used the significance level of 0.05 for two-sided testing.

## Results

We established a study cohort of 19042 patients with amyloidosis and a comparison cohort of 19042 people without the disease. The amyloidosis and comparison cohorts had similar distributions of sex and age, with a mean age of 55-years and a higher number of males in the studied populations (Table 1). The comparison cohort exhibited higher prevalence than the amyloidosis cohort in terms of most comorbidities, except for hypertension and hyperlipidemia. Compared to comparison cohort, a higher proportion of patients with amyloidosis were diagnosed with psychiatric disorder. The mean follow-up period was 6.97 (SD = 3.39) and 6.25 (SD = 3.34) years in the amyloidosis and comparison cohorts, respectively.

**Table 1:** Baseline characteristics in patients with and without amyloidosis.

Variable	Amyloidosis				p-value
	No (n = 19042)		Yes (n = 19042)		
	n	%	n	%	
**Sex**					0.003
Woman	9054	47.60	8760	46.00	
Man	9988	52.50	10282	54.00	
**Age, years**					0.001
< 49	7913	41.60	7280	38.20	
50–64	5411	28.40	6612	34.70	
≥ 65	5718	30.00	5150	27.10	
Mean ± SD ^a^	54.9	17.40	54.5	15.80	0.02
**Comorbidity**					
Hypertension	8987	47.20	9049	47.50	0.52
Diabetes mellitus	5454	28.60	5384	28.30	0.430
Hyperlipidemia	7298	38.30	7663	40.20	<0.001
Atrial fibrillation	4501	23.60	2578	13.50	<0.001
CAD	5469	28.70	4135	21.70	<0.001
Sleep disorders	6793	35.70	6322	33.20	<0.001
Anxiety	4752	25.00	3334	17.50	<0.001
Stroke	2329	12.20	1518	7.97	<0.001
COPD	3942	20.70	3440	18.10	<0.001
**Outcome**					
Psychiatric Disorder	1228	6.45	1495	7.85	<0.001
**Mean follow-up, (SD)^a^**	6.25	3.34	6.97	3.39	<0.001

Chi-Square test; ^a^*t*-test.

Table 2 shows amyloidosis and other risk factors for psychiatric disorder in the study population. The psychiatric disorder incidence was higher in the amyloidosis than in the comparison cohort (11.3 vs. 10.3 per 1000 person-years, crude HR = 1.10, 95% CI 1.02–1.19), with adjusted HR (aHR) of 1.26 (95% CI 1.16–1.36). The aHR of psychiatric disorder development was a 0.89-fold lower (95% CI 0.82‒0.96) for men as opposed to women. Compared to aged ≤ 49 group, the risk of psychiatric disorder was 1.65-fold increase (95% CI 1.45‒1.88) with aged 50‒64 group, and was 6.24-fold increase (95% CI 5.52‒7.04) with aged ≥ 65 group. The risk of developing psychiatric disorder was higher in patients with hypertension (adjusted HR = 1.27; 95% CI 1.16‒1.40), sleep disorders (adjusted HR = 1.33; 95% CI 1.23‒1.44), anxiety (adjusted HR = 1.91; 95% CI 1.75‒2.07), and stroke (adjusted HR = 2.01; 95% CI 1.83‒2.21).

**Table 2:** Cox model-measured hazard ratios and 95% confidence intervals of psychiatric disorder associated with amyloidosis and covariates or not.

Characteristics	Event			Crude			Adjusted		
	n	PY	IR^a^	HR	(95%CI)	p-value	HR	(95%CI)	p-value
**Status**									
Non-Amyloidosis	1228	119103	10.30	1	(Reference)	‒	1	(Reference)	‒
Amyloidosis	1495	132808	11.30	1.1	(1.02, 1.19)	0.01	1.26	(1.16, 1.36)	<0.001
**Sex**									
Woman	1292	123256	10.50	1	(Reference)	‒	1	(Reference)	‒
Man	1431	128655	11.10	1.06	(0.98, 1.14)	0.160	0.89	(0.82, 0.96)	0.002
**Age, years**									
< 49	449	113971	3.94	1	(Reference)	‒	1	(Reference)	‒
50–64	606	84197	7.20	1.83	(1.62, 2.07)	<0.001	1.65	(1.45, 1.88)	<0.001
≥ 65	1668	53743	31.00	7.89	(7.11, 8.77)	<0.001	6.24	(5.52, 7.04)	<0.001
**Comorbidity**									
Hypertension									
No	871	138858	6.27	1	(Reference)	‒	1	(Reference)	‒
Yes	1852	113053	16.40	2.60	(2.40, 2.82)	<0.001	1.27	(1.16, 1.40)	<0.001
Diabetes mellitus									
No	1670	182236	9.16	1	(Reference)	‒	1	(Reference)	‒
Yes	1053	69675	15.10	1.65	(1.52, 1.78)	<0.001	1	(0.92, 1.09)	0.99
Hyperlipidemia									
No	1532	148800	10.30	1	(Reference)	‒	1	(Reference)	‒
Yes	1191	103111	11.60	1.13	(1.05, 1.22)	0.002	0.73	(0.67, 0.79)	<0.001
Atrial fibrillation									
No	2055	212033	9.69	1	(Reference)	‒	1	(Reference)	‒
Yes	668	39878	16.80	1.71	(1.56, 1.86)	<0.001	0.88	(0.80, 0.97)	0.007
CAD									
No	1692	194882	8.68	1	(Reference)	‒	1	(Reference)	‒
Yes	1031	57029	18.10	2.07	(1.91, 2.23)	<0.001	1.06	(0.97, 1.16)	0.19
Sleep disorders									
No	1404	162096	8.66	1	(Reference)	‒	1	(Reference)	‒
Yes	1319	89815	14.70	1.70	(1.58, 1.84)	<0.001	1.33	(1.23, 1.44)	<0.001
Anxiety									
No	1692	198551	8.52	1	(Reference)	‒	1	(Reference)	‒
Yes	1031	53360	19.30	2.27	(2.10, 2.45)	<0.001	1.91	(1.75, 2.07)	<0.001
Stroke									
No	2111	233304	9.05	1	(Reference)	‒	1	(Reference)	‒
Yes	612	18607	32.90	3.58	(3.27, 3.91)	<0.001	2.01	(1.83, 2.21)	<0.001
COPD									
No	1980	205573	9.63	1	(Reference)	‒	1	(Reference)	‒
Yes	743	46338	16.00	1.66	(1.53, 1.81)	<0.001	0.93	(0.85, 1.01)	0.09

^a^ IR, Incidence Rate, per 1,000 person-years.

HR, Hazard Ratio; CI, Confidence Interval; Adjusted HR, Adjusted for age, sex, and comorbidities in the Cox proportional hazards model.

The incidence rate of senile and pre-senile organic psychotic conditions in amyloidosis cohort was 4.5 per 1,000 person-year and that in comparison cohort was 3.95 per 1,000 person-years (Table 3). After controlling for other confounders, amyloidosis cohort had a higher risk of senile and pre-senile organic psychotic conditions than comparison cohort (adjusted HR = 1.38; 95% CI 1.22‒1.57). Compared to comparison cohort, patients with amyloidosis had 1.16-fold (95% CI 1.01‒1.32) risk of other organic psychotic conditions. Patients with amyloidosis had 1.26-fold risk of bipolar affective disorder than comparison cohort (95% CI 1.10‒1.45).

**Table 3:** Incidences and hazard ratios of psychiatric disorder for individuals with and without amyloidosis.

Outcome (ICD-9-CM)	Non-Amyloidosis		Amyloidosis		Crude			Adjusted		
	Events	IR^a^	Events	IR^a^	cHR	(95% CI)	p-value	aHR	(95% CI)	p-value
Senile and presenile organic psychotic conditions (290)	471	3.95	597	4.5	1.15	(1.02, 1.30)	0.02	1.38	(1.22, 1.57)	<0.001
Alcoholic psychoses (291)	24	0.2	19	0.14	0.74	(0.41, 1.36)	0.33	0.73	(0.40, 1.35)	0.31
Drug psychoses (292)	15	0.13	5	0.04	0.31	(0.11, 0.85)	0.02	0.37	(0.13, 1.03)	0.06
Transient organic psychotic conditions (293)	82	0.69	93	0.7	1.02	(0.76, 1.37)	0.9	1.16	(0.86, 1.58)	0.33
Other organic psychotic conditions (294)	434	3.64	498	3.75	1.02	(0.89, 1.16)	0.8	1.16	(1.01, 1.32)	0.03
Schizophrenic disorders (295)	18	0.15	13	0.1	0.66	(0.32, 1.34)	0.25	0.72	(0.35, 1.49)	0.37
Bipolar affective disorder (296)	396	3.32	479	3.61	1.09	(0.95, 1.24)	0.22	1.26	(1.10, 1.45)	<0.001
Paranoid states (297)	11	0.09	20	0.15	1.67	(0.80, 3.48)	0.17	1.9	(0.89, 4.06)	0.1
Other nonorganic psychosis (298)	38	0.32	43	0.32	1.03	(0.66, 1.59)	0.91	1.07	(0.69, 1.66)	0.77

^a^ IR, Incidence Rate, per 1,000 person-years.

HR, Hazard Ratio; CI, Confidence Interval; Adjusted HR, Adjusted for age, sex, and comorbidities in the Cox proportional hazards model.

## Discussion

This research analyzed large sized amyloidosis population and showed significant worse psychiatric outcomes comparing to comparisons. The sample size of each group is big enough comparing to prior studies.

There are several novel strategies designed for different therapeutic purposes for treating amyloidosis recently;^9^, ^10^, ^11^, ^12^ however, data of different strategies are not available in such a real-world nationwide database registry study. These important and relevant information should be presented and is worthy to mention this in the current study.

Previous studies have established that the major determinants for stroke in patients with AF are their risk profiles (nowadays often indexed or referenced by the CHA2DS2-Vas score) and whether the therapeutic anticoagulants are used.^13^, ^14^, ^15^, ^16^ Interesting, stroke but not AF was the risk factor for psychiatric disorder among people with amyloidosis; implying that the possible mechanism of psychiatric illness might be through AF independent stroke in this specific group.

Some might argue that amyloidosis on psychiatric outcomes is fully spurious and random findings because neuropsychiatric connections and hypotheses for these findings could not be fully explained. Others could simply criticize that the patients were more ill and more sick patients, therefore, this psychiatric disorder might have been reserved for this type of patients ‒ this implicates many biases that cannot be answered in a non-randomized study such as physician and selection bias. Despite the efforts to demonstrate the relationship between amyloidosis and psychiatric disorders, the evidence presented is merely suggestive and open to question. An alternative approach would be more suitable since the findings presented as those of a preliminary study and deserved further validated.

Among different psychiatric disorders, risks of senile and pre-senile organic psychotic conditions, bipolar affective disorder and other organic psychotic conditions were higher among people with amyloidosis. Such information might be a breakthrough finding for the health care providers so that further modifying procedures could be instituted timely to avoid the unexpected adverse psychiatric events.

With the mechanistic effects were proposed previously, detailed pathways remained incompletely understood. In addition, the results are association rather than causal relation.^17^ Despite the conclusions for this study might be of potential interesting, collection of further data with longer time interval with prospective design, and the hypothetical mechanism of increasing psychiatric disorders risk by amyloidosis deserves further exploration.

### Limitations

This study suffers several weaknesses, especially the coding for “psychiatric disorders” is likely to be underdiagnosed in the NHIRD. Similarly, the diagnosis of various comorbidities is also questionable by using only relevant ICD codes. Much attempts have been made to validate the diagnosis of “psychiatric disorders” and other codes. Therefore, there is considerable agreement between ICD clinical diagnosis and criteria-based diagnosis of psychiatric disorders and amyloidosis. Furthermore, data on biochemistry profiles, that hold prognostic relevance for this population are not provided at all, despite groups being frequency matched for common comorbidities. In several experts’ opinion, studies regarding amyloidosis should be designed prospectively despite the conclusion was drawn by retrospective and observational data. However, a sample size of 19042+19042 participants is far enough “large number of patients” as a standard of an observational study. The adjustment statistical methods were applied to overcome the limitations. Concerning the symptom of psychiatric disorders is complex and it is often difficult to diagnose; hence, collecting the subjects from a national health insurance database might be a reasonable approach.

## Conclusions

We demonstrated that the risk of psychiatric disorders was significantly higher in patients with amyloidosis than those without amyloidosis.

## Clinical trial number

Not applicable.

## Human ethics

This study is in accordance with the Declaration of Helsinki.

## Ethical approval

This study was approved by the Research Ethics Committee of China Medical University and Hospital (CMUH111-REC2-109(CR-1)).

## Consent to participate

Not required.

## Consent for publication

Not required.

## Disclaimer

The funders had no role in the study design, data collection and analysis, the decision to publish, or the preparation of the manuscript.

## Authors’ contributions

All authors contributed to the manuscript. All were involved in the design of the study, collected the data, performed the statistical analysis, and wrote the manuscript, and all authors were involved in the final approval of the manuscript. The authors declare that all data were generated in-house and that no paper mill was used.

## Funding

This study is supported in part by China Medical University (CMU112-ASIA-08). No additional external funding was received for this study.

## Data availability

Data are available upon reasonable request.

## Declaration of competing interest

The authors declare no conflicts of interest.
